# Cardiac sympathetic activity and lethal arrhythmic events: insight into bell-shaped relationship between ^123^I-*meta*-iodobenzylguanidine activity and event rates

**DOI:** 10.1186/s13550-024-01131-4

**Published:** 2024-07-21

**Authors:** Kenichi Nakajima, Tomoaki Nakata, Takahiro Doi, Derk O. Verschure, Viviana Frantellizzi, Maria Silvia De Feo, Hayato Tada, Hein J. Verberne

**Affiliations:** 1 Department of Nuclear Medicine/Functional imaging and Artificial Intelligence, Kanazawa University, 13-1 Takara-machi, Kanazawa, 920-8640 Japan; 2 Department of Cardiology, Hakodate-Goryoukaku Hospital, Hakodate, Japan; 3 Department of Cardiology, Teine Keijinkai Hospital, Sapporo, Japan; 4 Department of Cardiology, Zaans Medical Center, Zaandam, The Netherlands; 5 Department of Radiological Sciences, Oncology and Anatomo-Pathology, Sapienza - University of Rome, Rome, Italy; 6 Department of Cardiology, Kanazawa University, Kanazawa, Japan; 7 Department of Radiology and Nuclear Medicine, Amsterdam University Medical Center, Location AMC, University of Amsterdam, Amsterdam, The Netherlands

**Keywords:** Sympathetic innervation imaging, Heart-to-mediastinum ratio, Cardiac device therapy, Machine-learning model

## Abstract

**Background:**

^123^I-*meta*-iodobenzylguanidine (*m*IBG) has been applied to patients with chronic heart failure (CHF). However, the relationship between ^123^I*-m*IBG activity and lethal arrhythmic events (ArE) is not well defined. This study aimed to determine this relationship in Japanese and European cohorts.

**Results:**

We calculated heart-to-mediastinum (H/M) count ratios and washout rates (WRs) of 827 patients using planar ^123^I-*m*IBG imaging. We defined ArEs as sudden cardiac death, arrhythmic death, and potentially lethal events such as sustained ventricular tachycardia, cardiac arrest with resuscitation, and appropriate implantable cardioverter defibrillator (ICD) discharge, either from a single ICD or as part of a cardiac resynchronization therapy device (CRTD). We analyzed the incidence of ArE with respect to H/M ratios, WRs and New York Heart Association (NYHA) functional classes among Japanese (J; *n* = 581) and European (E; *n* = 246) cohorts. We also simulated ArE rates *versus* H/M ratios under specific conditions using a machine-learning model incorporating 13 clinical variables. Consecutive patients with CHF were selected in group J, whereas group E comprised candidates for cardiac electronic devices. Groups J and E mostly comprised patients with NYHA functional classes I/II (95%) and II/III (91%), respectively, and 21% and 72% were respectively implanted with ICD/CRTD devices. The ArE rate increased with lower H/M ratios in group J, but the relationship was bell-shaped, with a high ArE rate within the intermediate H/M range, in group E. This bell-shaped curve was also evident in patients with NYHA classes II/III in the combined J and E groups, particularly in those with a high (> 15%) *m*IBG WR and with ischemic, but not in those with non-ischemic etiologies. Machine learning-based prediction of ArE risk aligned with these findings, indicating a bell-shaped curve in NYHA class II/III but not in class I.

**Conclusions:**

The relationship between cardiac ^123^I-*m*IBG activity and lethal arrhythmic events is influenced by the background of patients. The bell-shaped relationship in NYHA classes II/III, high WR, and ischemic etiology likely aids in identifying patients at high risk for ArEs.

## Background

The incidence of chronic heart failure (CHF) has increased to the point where it is regarded as a pandemic, and is considered as a global health priority [1]. Estimates indicate that 1.3 million people in Japan will experience CHF by 2030 [2]. Timely and accurate diagnoses of heart failure (HF) have become essential to prevent delays in diagnosis and a consequently increased risk for events associated with HF such as potentially fatal arrhythmias. Main causes of death are end-stage congestive HF due to a pump failure and sudden fatal arrhythmic attack. The baseline characteristics of patients with CHF such as age, sex, underlying cardiac conditions and comorbidities widely vary among individuals who die due to HF or arrhythmias [3]. Moreover, pharmacological and non-pharmacological treatment strategies for these conditions have considerably advanced. The latter requires particularly careful consideration of cardiac electronical device intervention combined with intensive drug therapy. Conversely, a cardiac device might not be needed for patients at lower risk for lethal events. Appropriate risk stratification plays crucial roles in predicting clinical outcomes and in selecting the most effective strategies to treat HF.

^123^I-*meta*-iodobenzylguanidine activity (*m*IBG) is a robust predictor of lethal cardiac events among patients with CHF, and effective for risk stratification [4–6]. A low heart-to-mediastinum (H/M) ratio of cardiac ^123^I-*m*IBG activity is generally associated with a poorer prognosis, comprising overall cardiac death, HF progression, and lethal arrhythmic events (ArEs) [7, 8]. A long-term multicenter cohort study of a large Japanese population followed has revealed a clear, negative linear relationship between late H/M ratios and cardiac death [5]; that is, the incidence of cardiac death increases as H/M ratios decrease. More specifically, a bell-shaped curve of sudden cardiac death might correlate with H/M ratios [9–11]. This would imply that lethal events are likely to be more frequent with an intermediate H/M ratio and/or summed defect scores on tomographic images. However, considering the role of H/M ratios in predicting lethal ArE, a bell-shaped curve has not been consistent.

A risk model using ^123^I-*m*IBG incorporating multiple clinical factors can stratify patients with CHF and those with acute non-compensated HF [12, 13]. However, the ability of ^123^I-*m*IBG to stratify lethal arrhythmias has not been evaluated in detail. This might be partly explained by the limited clinical application of ^123^I-*m*IBG imaging that persists in Europe and the USA [14], whereas it is officially approved to assess a broad range of cardiac diseases in Japan.

This study aimed to determine the ability of ^123^I-*m*IBG to predict ArE by comparing the bell-shaped relationship between cardiac ^123^I-*m*IBG activity and (potentially) lethal ArEs in Japanese and European patients with CHF. We further characterized the bell-shaped relationship among Japanese and European patients using a machine-learning (ML)-based model [15] that was originally developed to differentiate between ArE and HFD in a Japanese population.

## Methods

### Patients

We retrospectively selected 827 patients with CHF based on clinical indications for ^123^I-*m*IBG cardiac scintigraphy. A Japanese cohort (group J) comprised 581 consecutive patients diagnosed with CHF. A European cohort (group E) consisted of 246 patients primarily studied in the context of cardiac devices such as implantable cardioverter defibrillators (ICDs) or cardiac resynchronization therapy with a defibrillator (CRTD). Patients with incomplete follow-up data at two years (censored alive within 2 years) were excluded. The ML model described below included 526 patients as a derivation group [15], which we excluded to avoid data overlap.

The ethics committee at Kanazawa University approved the study protocol (Approval number:1553/3020) and all participating institutions approved the multicenter data collection. The requirement for written informed consent was waived given the retrospective nature of the study. All data were transferred to a core laboratory in the form of a statistical table that was coded to render it innominate. The keys to these codes were available only to the principal investigators of the respective original databases.

### Definition of outcomes

Outcome events were classified as death resulting from HF (HFD) due to pump failure or lethal ArEs defined as sudden, arrhythmic, sustained ventricular tachycardia/fibrillation (VT/VF), cardiac arrest with resuscitation, and appropriate ICD discharge (either by single ICD or as part of a CRTD). However, the primary focus of the ^123^I-*m*IBG study in the E group was on arrhythmic events, and death resulting from end-stage HF were not documented in the database. We did not record death due to acute coronary syndromes in both groups.

### ^123^I-*m*IBG image acquisition and image analysis

Patients in the J and E groups were respectively administered intravenously with 111 and 185 MBq of ^123^I-*m*IBG, then early and late images were respectively acquired at 15–30 min and 4 h thereafter. Patient preparation and image acquisition complied with the respective European Association of Nuclear Medicine (EANM) recommendations and Japanese guidelines [16, 17]. Images were acquired in the anterior view over an interval of 5–10 min (128 × 128 or 256 × 256 matrix). Regions of interest (ROIs) were defined for the upper mediastinum and the heart. The H/M ratio was defined as the ratio between mean myocardial and mediastinal counts. Washout rates (WRs) were calculated using the early and late H/M ratios as: (early H/M - late H/M)/early H/M [18]. A standardized H/M ratio was obtained by adjusting medium-energy (ME) general-purpose collimator conditions to mitigate variations in H/M ratios due to differences in collimator types across facilities [19, 20].

### Other clinical variables

We analyzed the following variables: NYHA categorical classifications, left ventricular ejection fraction (LVEF; regardless of assessment type); B-type natriuretic peptide (BNP) and N-terminal (NT)-ProBNP values transformed into categories 0, 1, 2, 3 and 4 (representing < 40, 40−99, 100−199, 200−560 and > 560 pg/mL, and < 125, 125−399, 400−899, 900−4800 and > 4800 pg/mL, respectively [12, 15]), and the comorbidities of diabetes, dyslipidemia, hypertension, and coronary artery disease. The LVEF in the J group and in the Italian database was measured by two-dimensional echocardiography under stable conditions at each participating institution using a biplane modified Simpson method [21]. We also analyzed the LVEF derived from a combination of standard techniques at individual hospitals that included echocardiography, gated myocardial perfusion SPECT, or magnetic resonance imaging (MRI) also under stable conditions in another European database compiled in Amsterdam University [22, 23].

### Machine-learning (ML) model to distinguish lethal arrhythmic events

The relationship between the ^123^I-*m*IBG H/M ratio and the risk of mortality was assessed using an ML model as described [15]. This model was developed to distinguish lethal events between HFD and ArE (sudden cardiac death and appropriate ICD therapy) in patients with CHF. The model comprised 13 variables; age (y), sex (male/female; 1/0), NYHA functional classes 1‒4, eGFR (mL/min/1.73 m²), LVEF (%), hemoglobin (g/dL), ^123^I-*m*IBG late H/M ratio and WR (%), and BNP or NT-ProBNP levels (5 categorical values). Hemodialysis, ischemic etiology, hypertension, and diabetes are represented as yes (1) or no (0). Random forest (area under the curve [AUC] of receiver operating characteristic curve [ROC] = 0.92) and logistic regression (AUC = 0.90) were used to predict HFD [15]. However, we selected logistic regression for both ArE and HFD in the present study because it predicted ArEs optimally (AUC = 0.73). The probability of a severe event based on changes in H/M ratios was calculated using a stable logistic regression model. We predicted combined events as the sum of predicted ArE and HFD rates (namely, 1- predicted survival rate). Values were averaged when applying the model to particular conditions in groups J and E, assuming continuous variables as input. For instance, if 76% of the patients were male, the applied input value would be 0.76.

### Statistical analysis

Variables are expressed as means ± standard deviation (SD). Mean values between groups were compared using analyses of variance (ANOVA). Pairs of groups were compared using contingency table analyses with Pearson statistics for categorical variables. The accuracy of the model was determined by calculating the ROC AUC of the dataset. We determined relationships between events and risk levels derived from an ML-based model using Cochran Armitage trend tests. All data were statistically analyzed using JMP Pro 17 (SAS Institute Inc., Cary, NC, USA). Values with *P* < 0.05 were considered significant. We used Mathematica 14.0 (Wolfram Research Inc., Champaign, IL, USA) for modeling and simulation.

## Results

### Patient characteristics

**Fig. 1:**
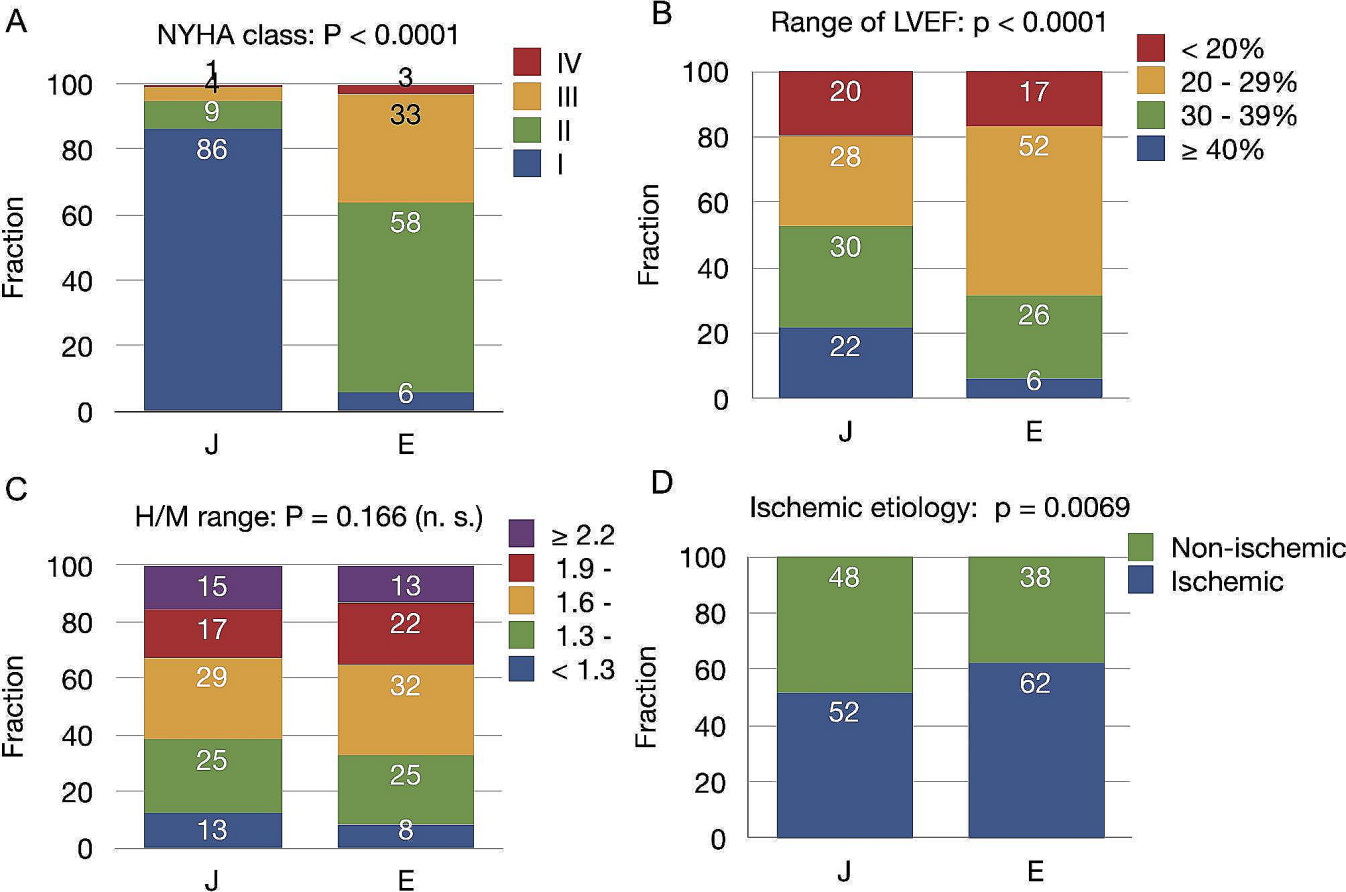
Fractions of variables for NYHA functional classes, ischemic etiology, and ranges of LVEF, ^123^I-*m*IBG H/M ratios in groups J (*n* = 581) and E (*n* = 246) E, European; H/M, heart-to-mediastinum; J, Japanese; LVEF, left ventricular ejection fraction

Table 1 and Fig. 1 summarize the patient characteristics. Groups J and E predominantly comprised patients with CHF and NYHA functional classes I/II (95%) and II/III (91%), respectively. The mean followup duration was 29.3 ± 13.4 and 31.5 ± 15.2 months for groups J and E, respectively. The ^123^I-*m*IBG early and late H/M ratios, and WRs did not significantly differ between the groups. The incidence of ischemic HF was slightly higher in group E than J (62.2% vs. 52.0%, *p* = 0.0069). The eGFR and proportions of hypertension were higher, and LVEF and Hb values were lower in group E, than J. Figure 1 shows the distributions of NYHA functional classes, LVEF, and HF etiology, as well as early and late H/M ratios. Cardiac devices (ICDs or CRTDs) were implanted in 121 (21%) and 178 (72%) patients in groups J and E, respectively.

**Table 1:** Demographics of Japanese (J) and European (E) patients

Group	Combined J and E	J	E	*P* (J vs. E)
*n*	827	581	246	
Entry criteria	Combined	Consecutive CHF determined by cardiac ^123^I-mIBG imaging	Candidates for cardiac electronic device treatment	
Age (y)	67.0 ± 12.1	68.1 ± 12.3	64.4 ± 11.4	< 0.0001
Male	639 (77%)	438 (75%)	201 (82%)	0.056
Mean followup (m)	29.9 ± 14.0	29.3 ± 13.4	31.5 ± 15.2	0.047
NYHA functional classes I, II, III, IV (%)	62%/23%/13%/2%	86%/9%/4%/1%	6%/58%/33%/3%	< 0.0001
Standardized early ^123^I-*m*IBG H/M ratio*	2.00 ± 0.43	1.99 ± 0.46	2.00 ± 0.37	0.95
Standardized late ^123^I-*m*IBG H/M ratio*	1.78 ± 0.42	1.77 ± 0.44	1.79 ± 0.37	0.46
^123^I-*m*IBG washout rate (%)	10.70 ± 9.6	11.0 ± 9.6	10.1 ± 9.6	0.21
eGFR (mL/min/1.73 m^2^)	52.4 ± 29.0	44.7 ± 25.5	77.5 ± 35.4	< 0.0001
LVEF (%)	30.3 ± 10.3	31.0 ± 10.8	28.8 ± 9.0	0.0025
Ischemic etiology	55%	52%	62%	0.0069
BNP/NT-proBNP grade	2.4 ± 1.3	2.2 ± 1.3	3.1 ± 1.1	< 0.0001
Hemoglobin (g/dL)	12.0 ± 2.4	12.3 ± 2.3	11.1 ± 2.4	< 0.0001
Hypertension (%)	55%	48%	70%	< 0.0001
Diabetes mellitus (%)	36%	36%	36%	0.95
Hemodialysis (%)	3%	4%	0%	0.001
ICD/CRTD	299 (36%)	121 (21%)	178 (72%)	< 0.0001

*Standardized to MEGP collimator status. BNP, B-type natriuretic peptide; CHF, chronic heart failure; eGFR, estimated glomerular filtration rate; ICD/CRTD, implantable cardioverter defibrillator/cardiac resynchronization therapy with defibrillator; LVEF, left ventricular ejection fraction; MEGP, medium-energy general purpose; *m*IBG, *meta*-iodobenzylguanidine; NT-proBNP, N-terminal pro b-type natriuretic peptide; NYHA, New York Heart Association

We compared patients with and without implanted ICD/CRTDs (Table 2). The LVEF, BNP/NT-ProBNP values, and the proportions of patients who were complicated by hypertension and diabetes were lower in group J with, than without implanted devices. The LVEF, late ^123^I-*m*IBG H/M ratio, proportion complicated by hypertension, Hb value and eGFR were lower, and WR and BNP/NT-ProBNP values were higher, among patients in group E who were implanted ICD/CRTDs. A comparison of J and E subgroups with ICD/CRTDs revealed that patients were older, had NYHA classes with better functional capability, lower BNP/NT-ProBNP and eGFR values, and a lower proportion of ischemic etiology and hypertension in group J than E.

**Table 2:** Comparison of Japanese (J) and European (E) patients with and without ICD/CRTD implantation

	J without ICD/CRTD	J with ICD/CRTD	*P*	E without ICD/CRTD	E with ICD/CRTD	*P*	P*
*n*	460	121		68	178		
Age	68 ± 13	69 ± 10	0.39	63 ± 12	65 ± 11	0.23	0.0018
Sex (Male, %)	341 (74%)	97 (80%)	0.17	60 (88%)	141 (79%)	0.1	0.84
NYHA class	1.2 ± 0.5	1.3 ± 0.7	0.12	2.3 ± 0.7	2.3 ± 0.6	0.89	< 0.0001
(I/II/III/IV%)	87%/8%/4%/1%	83%/9%/6%/2%	0.22	13%/44%/40%/3%	4%/63%/30%/3%	0.0067	< 0.0001
LVEF (%)	32 ± 10	27 ± 11	< 0.0001	32 ± 10	27 ± 8	0.0007	0.95
BNP/NT-ProBNP category	2.3 ± 1.3	2.0 ± 1.2	0.015	2.6 ± 1.1	3.3 ± 1.0	0.0001	< 0.0001
^123^I-*m*IBG late H/M ratio^†^	1.8 ± 0.4	1.7 ± 0.4	0.58	1.9 ± 0.4	1.7 ± 0.4	0.001	0.96
^123^I-*m*IBG WR (%)	11 ± 10	11 ± 9	0.56	6 ± 9	12 ± 9	< 0.0001	0.87
Hemoglobin (g/dL)	12.3 ± 2.3	12.5 ± 2.0	0.27	12.5 ± 1.7	10.5 ± 2.4	< 0.0001	< 0.0001
eGFR (mL/min/1.73 m^2^)	45 ± 26	45 ± 25	0.83	85 ± 36	65 ± 24	< 0.0001	< 0.0001
Hemodialysis (*n* %)	21 (5%)	4 (3%)	0.54	0 (0%)	0 (0%)	-	0.015
Ischemic etiology (*n* %)	242 (53%)	60 (50%)	0.55	42 (62%)	111 (62%)	0.93	0.029
Hypertension (*n* %)	233 (51%)	47 (39%)	0.021	55 (80%)	117 (66%)	0.021	< 0.0001
Diabetes (*n* %)	177 (38%)	32 (26%)	0.014	25 (37%)	64 (36%)	0.9	0.084

*Between J and E with ICD/CRTD implants. ^†^Standardized to MEGP collimator status. BNP, B-type natriuretic peptide; eGFR, estimated glomerular filtration rate; ICD/CRTD, implantable cardioverter defibrillator/cardiac resynchronization therapy with defibrillator; LVEF, left ventricular ejection fraction; *m*IBG, *meta*-iodobenzylguanidine; NT-proBNP, N-terminal pro b-type natriuretic peptide; NYHA, New York Heart Association; WR, washout rate

### Probability of event risk and two-year outcomes

The probability of HFD in group J predicted by the ML model was 10.1% and the actual value was 14.5% (Table 3). The predicted probability of ArEs according to the ML model and the actual values were 8.5% and 7.9%, respectively. This indicated good prediction capacity of the ML model. The combined probability of HFD and ArEs in group E predicted by the ML model vs. the actual value was 34.7% vs. 33.7%. However, HFD did not occur in group E.

**Table 3:** Documented outcomes of Japanese (J) and European (E) patients

	J	E	Probability
*n*	581	246	
End-stage heart failure death	84 (14.5%)	-	
Lethal arrhythmic events + appropriate ICD/CRTD therapy	46 (7.9%)	83 (33.7%)	< 0.0001
Appropriate ICD/CRTD therapy	12	32	
Sudden cardiac death	3	18	
Sustained VT/VF	21	25	
Resuscitation after CPA	5	8	
CPA death	5	0	
Survived	451 (77.6%)	163 (66.3%)	0.0006

CPA, cardiopulmonary arrest; ICD/CRTD, implantable cardiac defibrillator/cardiac resynchronization therapy with defibrillator; VF, ventricular fibrillation; VT, ventricular tachycardia

### ^123^I-*m*IBG H/M ratio and incidence of lethal arrhythmic events

**Fig. 2:**
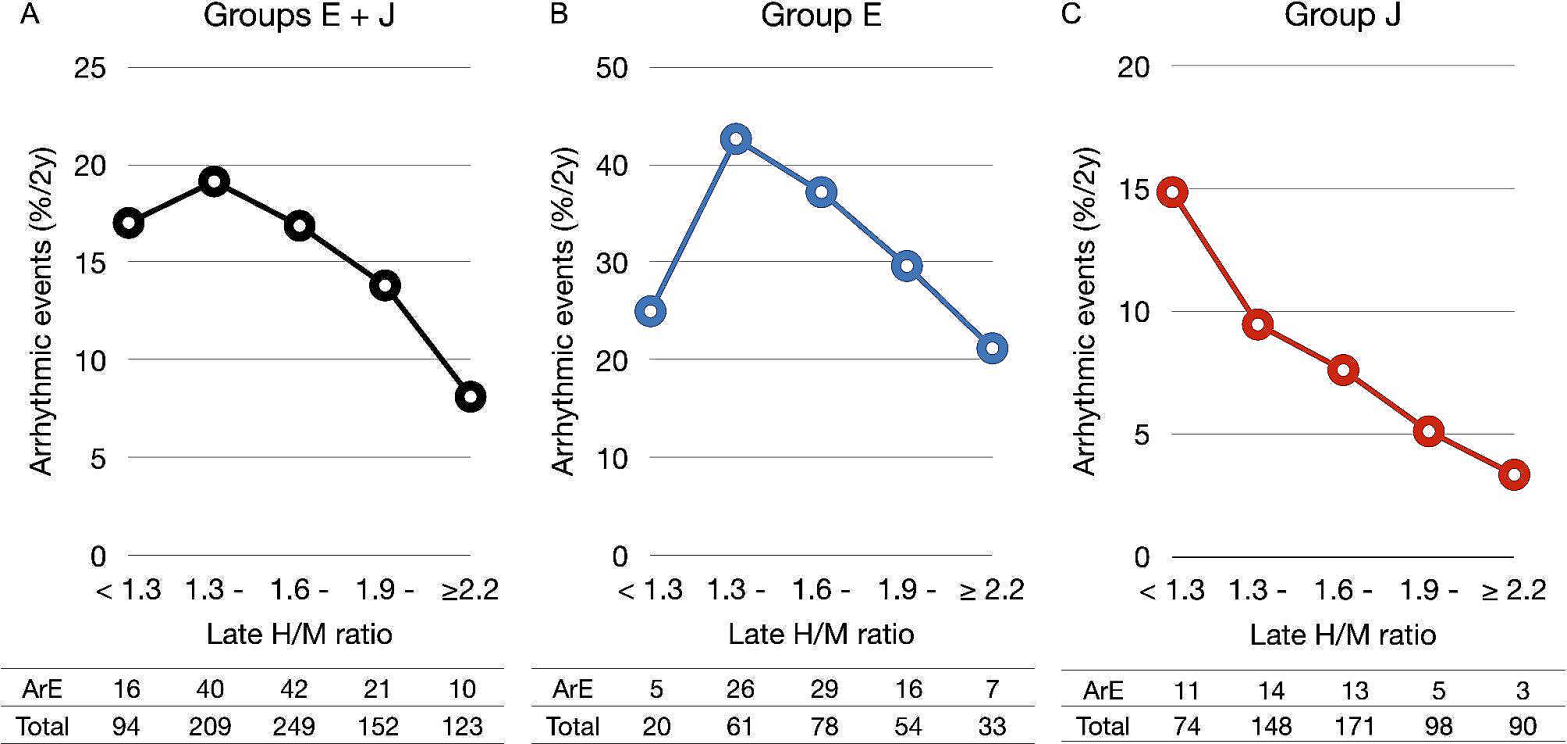
Incidence of lethal arrhythmic events **Panels A-C**: European (E) plus Japanese (J), European, and Japanese patients, respectively. Actual numbers of patients and events per subgroup are shown below each graph

Figure 2 shows correlations between the late H/M ratio and ArE during followup. Table 3 shows a substantially higher incidence of ArE in group E than J. However, ArEs were associated with a decreased late H/M ratio in group J; the event rate was maximal in the lowest late H/M ratio category (< 1.3) (Fig. 2C). In contrast, we found a bell-shaped relationship in group E. That is, the highest ArE was within the mid-range of late H/M ratio categories from 1.3 to 1.9 (Fig. 2B).

**Fig. 3:**
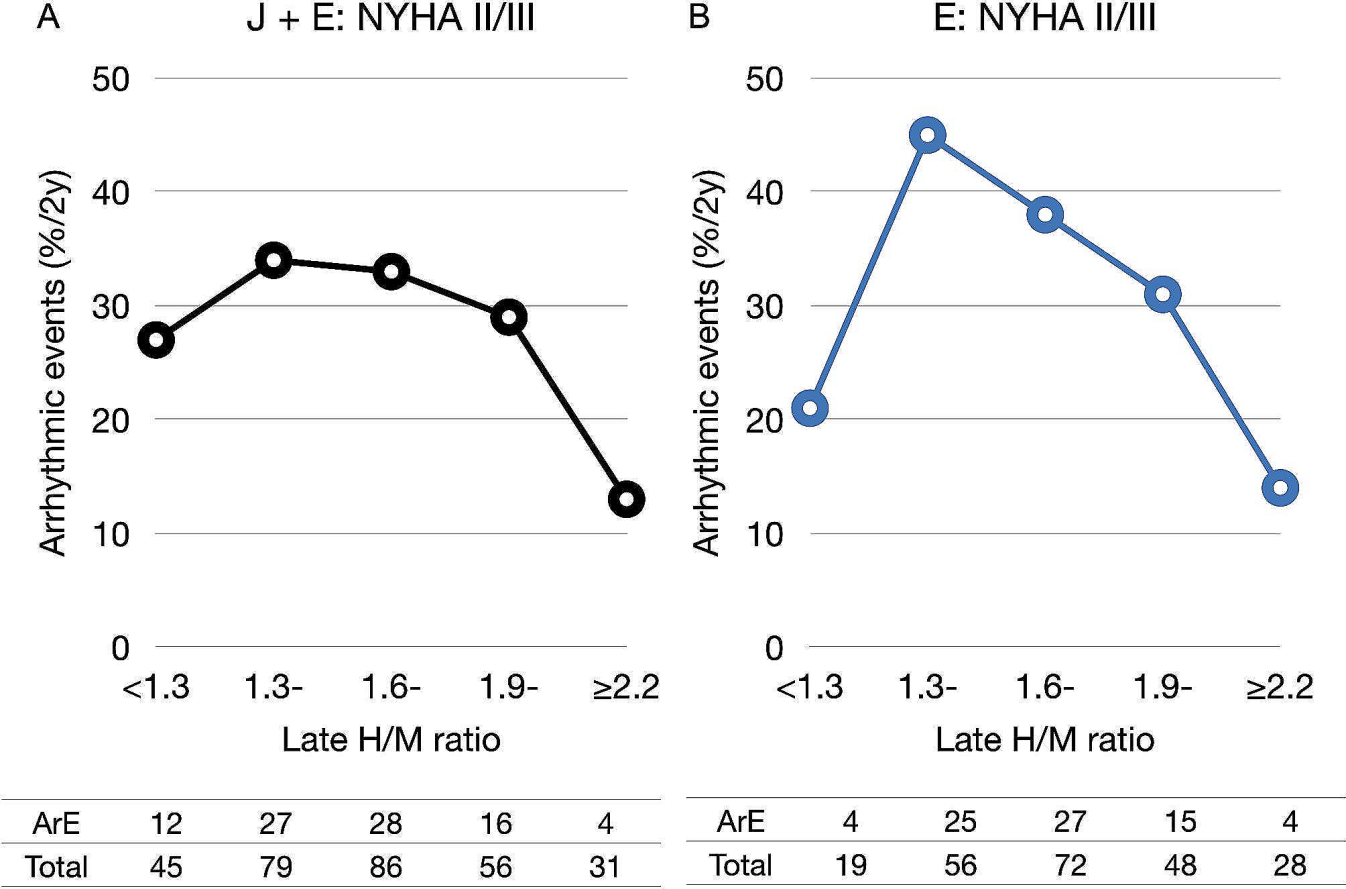
Lethal arrhythmic event rates and late ^123^I-*m*IBG H/M ratios in patients with NYHA classes II and III **A** and **B**, derived from E and J combined databases and group E database, respectively. H/M, heart-to-mediastinum

The bell-shaped relationship between ArE and late H/M categories was even clearer in NYHA functional classes II and III (Fig. 3). The incidence of ArEs in the H/M range from 1.3 to 1.9 was higher not only in group E, but also in the group with combined NYHA functional classes II and III. This effect was primarily driven by the European data because incidence of ArEs was low (only 0‒8) for each range of H/M ratios in group J with NYHA classes II and III.

### Relation between ^123^I-*m*IBG WRs and arrhythmic events

**Fig. 4:**
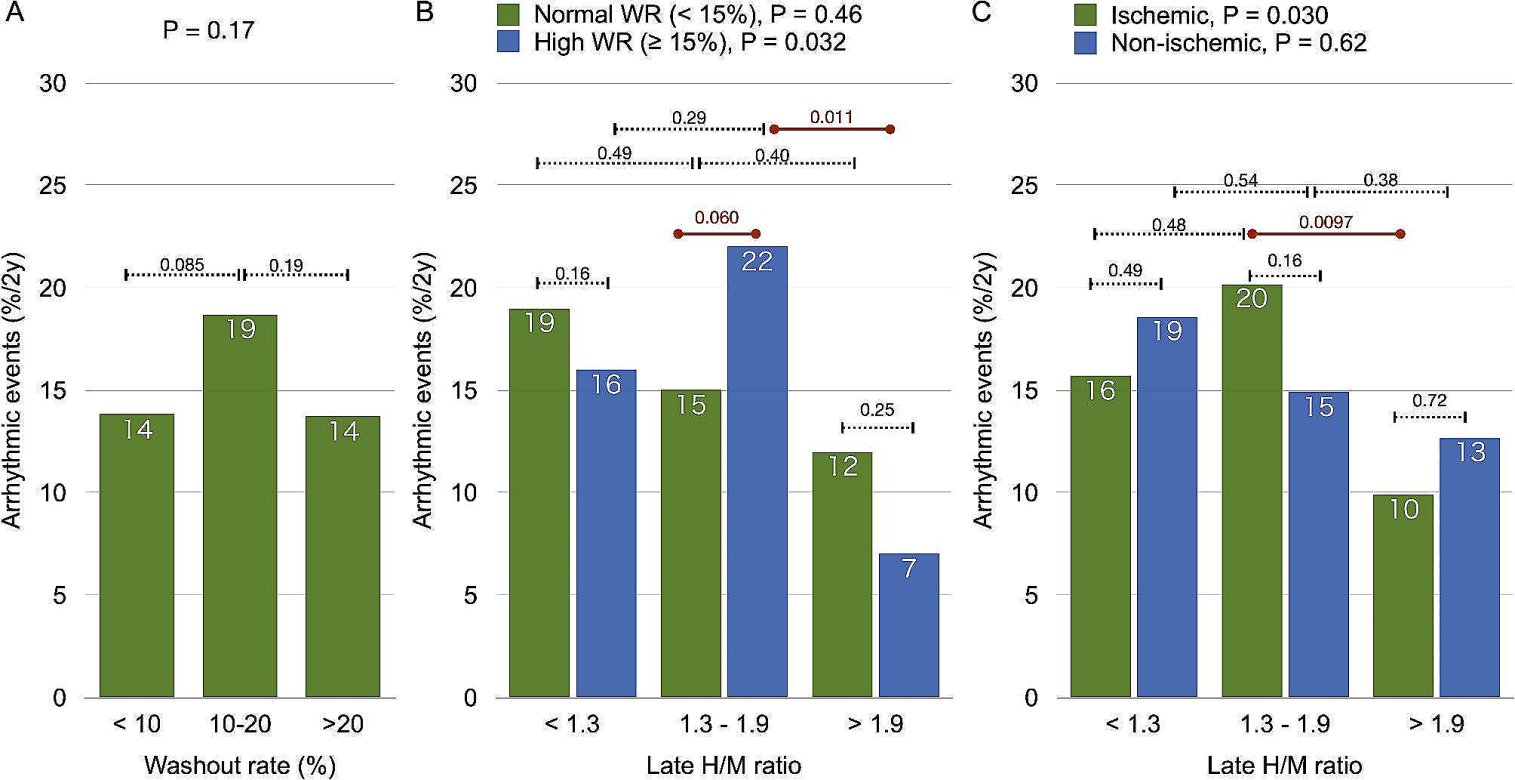
Relationships between late H/M ratios and lethal arrhythmic events **A**: lethal arrhythmic event incidence associated with three washout categories. **B**: normal (green, *n* = 520) and increased (blue, *n* = 307) WRs. **C**: ischemic (green, *n* = 455) and non-ischemic (blue, *n* = 372) etiologies. H/M, heart-to-mediastinum; WR, washout rates

The correlation between ^123^I-*m*IBG WRs and the incidence of ArEs was also bell-shaped in the combined groups J and E, with a maximal event rate for WRs between 10−19%. Previous studies showed a normal upper limit of the ^123^I-*m*IBG WR at 14.2% [18]. For the current analyses we therefore categorized patients as having increased (≥ 15%) or normal WRs (< 15%). Figure 4 shows that ArE rate increased as the H/M ratio decreased in patients with normal WRs. Conversely, the peak ArE incidence was 22% in the intermediate H/M range in patients with increased WRs (H/M 1.3–1.9 vs. > 1.9, *p* = 0.011). The ArE rate was higher among patients in the intermediate H/M range with high, than normal WRs (*p* = 0.060). A bell-shaped curve was notably absent among patients with non-ischemic, compared with ischemic etiology (Fig. 4C).

### Arrhythmic events predicted by ML model

**Fig. 5:**
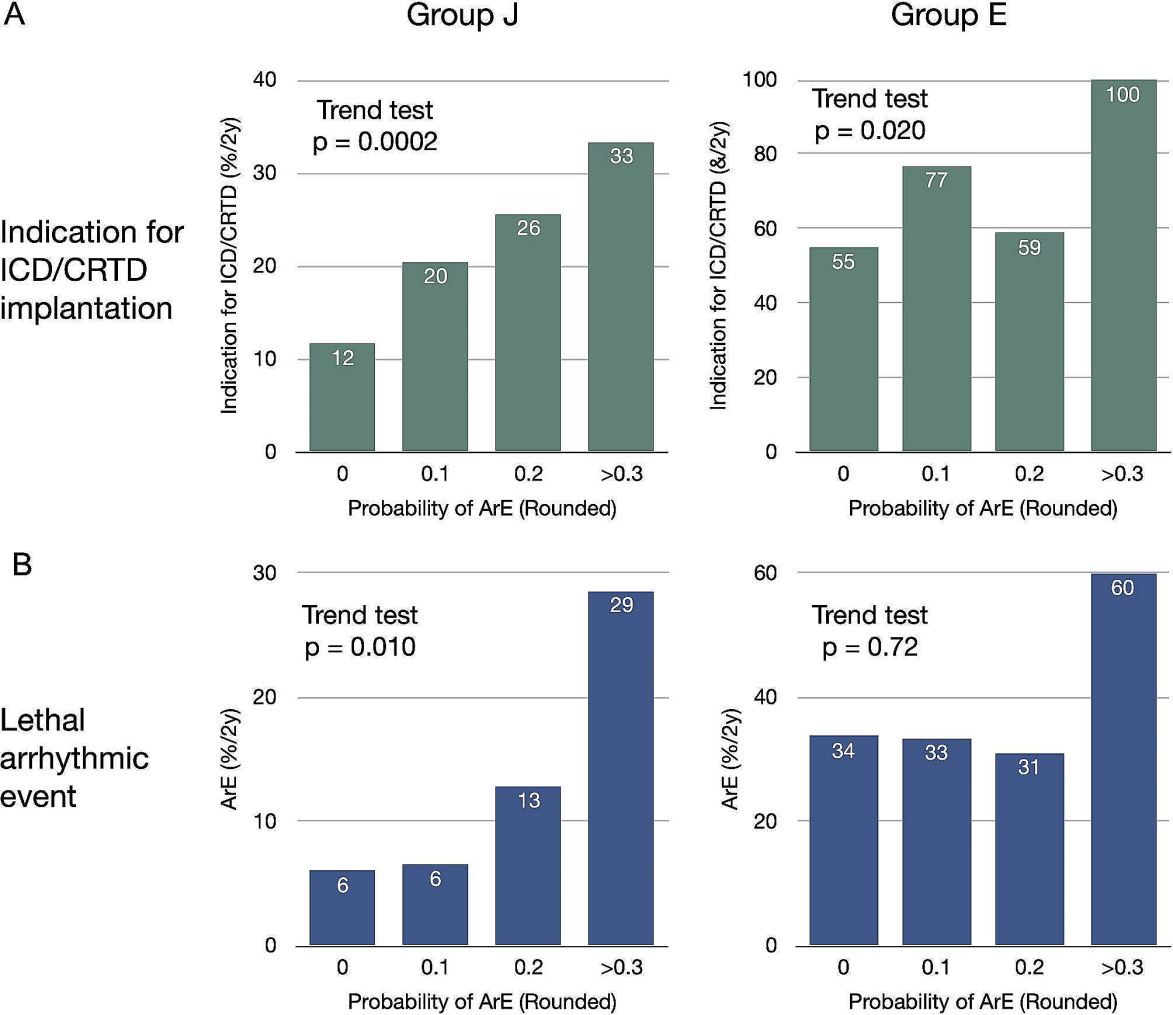
Indications for ICD/CRTD implantation (**A**) and predicted risk of lethal ArEs (**B**) in groups J and E Probability of ArE is rounded to one decimal place. ArEs, arrhythmic events. ICD/CRTD, implantable cardioverter defibrillator/cardiac resynchronization therapy device

We assessed the ability of the ML model to determine indications for ICD/CRTD implantation. We found a close correlation between risk and incidence of cardiac device implantation in group J (Fig. 5A, Cochran Armitage trend test *p* = 0.0002) and a positive trend in group E (*p* = 0.020). The AUCs were 0.721 (*p* < 0.0001) for combined HFD and ArE events and 0.643 (*p* = 0.0007) for ArE alone in group J according to the ML model. The predicted risk was consistent with actual ArEs (*p* = 0.010) in group J (Fig. 5B). In contrast, the AUC for ArE in group E was poor (0.472, *p* = 0.86), and the trend test with respect to risk level vs. ArE rate was not significant, primarily due to misclassified low-risk patients.

**Fig. 6:**
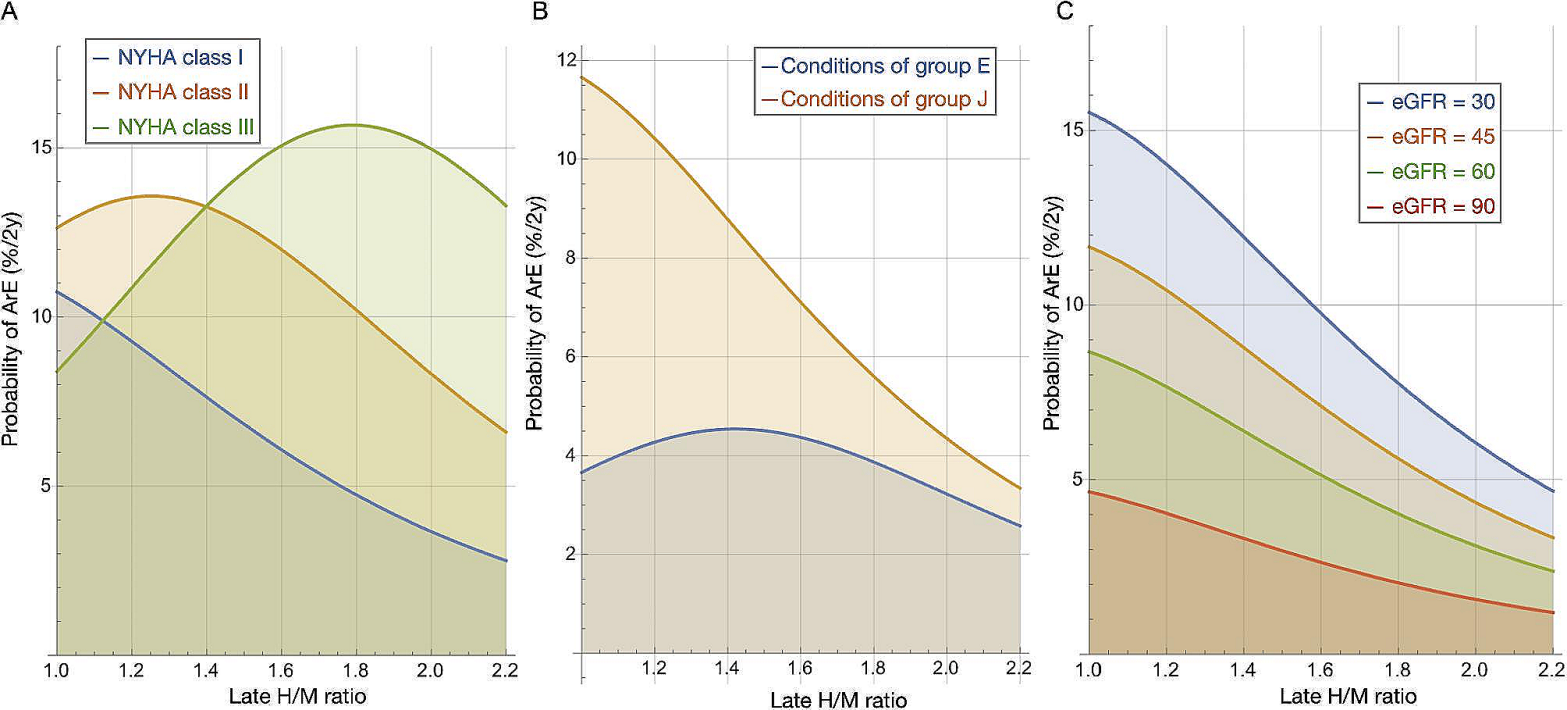
Lethal arrhythmic event rates vs. late H/M ratios simulated by ML model [15] **A**: effects of NYHA functional class on lethal ArEs. **B**: average values derived from conditions specific to groups E and J. Input values were age, 68 y, sex, 0.76; eGFR, 45; LVEF, 31%; ischemia, 0.5; hemoglobin, 12; BNP value, 2.2; hypertension, 0.52; diabetes, 0.64; without hemodialysis, as in European cohort. **C**: Effects of eGFR on lethal ArEs. ArEs, arrhythmic events; BNP, B-type natriuretic peptide; eGFR, estimated glomerular filtration rate; H/M, heart-to-mediastinum; ML, machine learning; NYHA, New York Heart Association

**Fig. 7:**
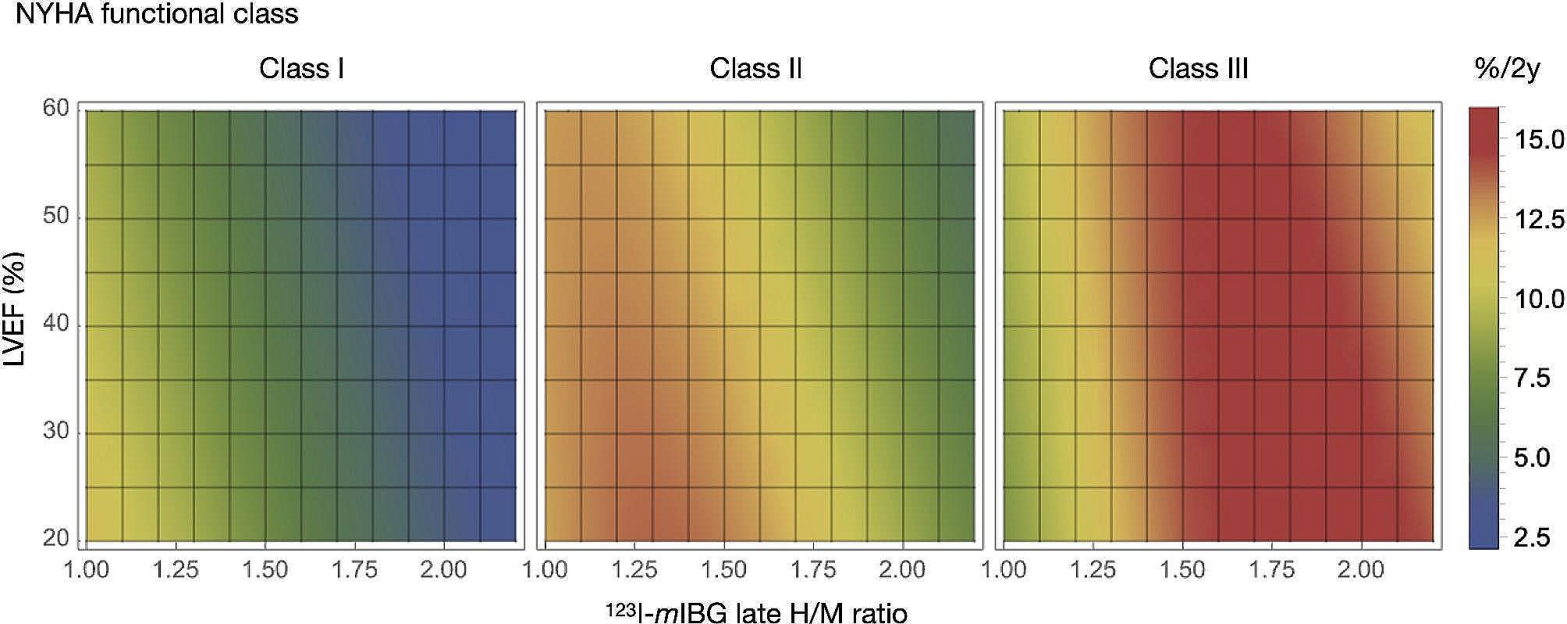
Density plots of arrhythmic event probability for left ventricular ejection fraction vs. H/M ratios in patients with NYHA classes I, II, and III H/M, heart-to-mediastinum; ML, machine learning; NYHA, New York Heart Association

We analyzed possible factors to explain the bell-shaped curve using a multivariable ML model [15]. Assuming that all variables were continuous numbers and that the values were averaged, we plotted ArE rates against late H/M ratios (Fig. 6). The curve was notably bell-shaped for NYHA class II/III, and the peak shifted rightward as the NYHA class increased. The curve was bell-shaped exclusively in group E (Fig. 6B). However, whereas the eGFR was lower in group J than E, its relationship with ArE was not bell-shaped (Fig. 6C). The effects of the H/M ratio and LVEF on ArEs were plotted two-dimensionally against each NYHA class (Fig. 7). The risk of ArEs was generally increased when both the H/M ratio and LVEF were low. The simulated graph for NYHA class II/III, shows a high risk of ArEs in the intermediate H/M range. The effects of the H/M ratio were more dominant than LVEF in estimating risk of ArEs.

## Discussion

Although the prognostic value of cardiac ^123^I-*m*IBG derived parameters in HF patients has been determined, evidence about lethal arrhythmia-related events remains inconclusive. A low H/M ratio has consistently correlated with unfavorable prognoses in terms of HF-related mortality since the 1990s. Despite robust evidence of the ^123^I-*m*IBG H/M ratio as an independent prognostic factor, a reduced ^123^I-*m*IBG H/M ratio has not necessarily been identified as a significant predictor of lethal ArEs in patients with CHF. The present findings revealed a bell-shaped, rather than a linear correlation between the H/M ratio and lethal ArEs. That is, the risk of lethal ArEs is most likely to increase when the ^123^I-*m*IBG H/M ratio is intermediately reduced in patients with NYHA functional classes II and III CHF or when the ^123^I-*m*IBG WR is increased (≥ 15%).

### Differences in patient characteristics between Japan and Europe

The present findings revealed variations in patient characteristics. This should be considered so that cardiac ^123^I-*m*IBG imaging and H/M ratios can assess the risk of lethal ArEs more effectively among patients with CHF. The health insurance system in Japan has officially covered the application of clinical ^123^I-*m*IBG imaging to a wide range of cardiac diseases since 1992. Thus, ^123^I-*m*IBG imaging has become a versatile diagnostic approach for various conditions. The Japanese Circulation Society specifically acknowledges the prognostic implications of ^123^I-*m*IBG imaging findings in patients with HF independently of ischemic background [24]. Consequently, overall cardiac mortality and specific risk for lethal ArEs (including sudden death) during the long-term clinical course of HF have been assessed by ^123^I-*m*IBG imaging [4–11]. The AdreView Myocardial Imaging for Risk Evaluation in Heart Failure (ADMIRE-HF) study found that ^123^I-*m*IBG imaging was associated with risk of lethal arrhythmic events in patients with NYHA functional class II/III and LVEF < 35% [4]. This raised the question as to whether ^123^I-*m*IBG imaging could contribute to more appropriate use of cardiac device therapy in CHF. Indeed, ^123^I-*m*IBG scintigraphy appears helpful in selecting patients with CHF who might not benefit from ICD implantation [9]. Part of that cohort was included in the present study, and the selection of patients with CHF might have resulted in bias due to lack of information about cardiac deaths due to pump failure in group E.

### Bell-shaped correlation between ArE risk and impaired cardiac sympathetic innervation

Despite several investigations, a relationship between the H/M ratio of ^123^I-*m*IBG activity and lethal ArEs has not been established. A Japanese study of patients with implanted ICDs (*n* = 54) found that BNP values, any medication, and reduced cardiac ^123^I-*m*IBG activity (H/M ratio) were significant independent predictors of appropriate ICD discharge over a 15-month period with a cutoff of 1.95 and an AUC of 0.683 [7]. Another Japanese multivariable analysis (*n* = 106), found that high myocardial ^123^I-*m*IBG WRs were more potent predictors of sudden cardiac death than late H/M or LVEF [8].

On the other hand, a European multicenter study of ICD therapy in patients with CHF found that ICD therapy was appropriate only within a 1.40‒2.19 range of late H/M ratios [9]. However, ICD therapy might have not been indicated for patients with an extremely low (< 1.40) or normal H/M ratio (> 2.2) in that study. An Italian study (*n* = 170) similarly identified early summed SPECT scores as the sole independent predictor of ArEs [10]. However, when considering the late H/M ratio, the incidence of appropriate therapy was high in the intermediate range of 1.2‒1.6.

An ADMIRE-HF sub-study (*n* = 622) found that intermediate, abnormal, summed defect scores derived from SPECT images were associated with an increased likelihood of ArEs [11]. However, not all SPECT-derived data were available in our study. Regional abnormalities in cardiac sympathetic innervation can be identified by SPECT imaging, which might be more effective than planar images in terms of localizing an arrhythmogenic injured myocardium to predict ArE including sudden cardiac death [25]. However, regional analyses with subsequent defect or ^123^I-*m*IBG/perfusion mismatch scores are limited when patients have extensively reduced ^123^I-*m*IBG activity. The application of SPECT methods holds promise but requires refinement and integration with appropriate normal databases.

When relationships are bell-shaped, statistical dichotomous or logistic analyses might yield insignificant results regarding the role of the H/M ratio for predicting ArEs. However, when the incidence of events was plotted against the H/M range, the trend became bell-shaped. The present findings of a bell-shaped correlation between lethal ArE risk and intermediately reduced ^123^I-*m*IBG H/M ratios particularly in patients with NYHA functional class II/III CHF or with increased WRs in both cohorts were in line with overall previous results.

### Reasons for bell-shaped curves for arrhythmic event risks

Details of the mechanism behind the bell-shaped correlation between lethal ArE risk and ^123^I-*m*IBG H/M ratio remain vague, but some potential explanations can be considered. An important point is that disrupted sympathetic innervation is associated with arrhythmias. However, a sufficient amount of myocardium at risk is also important. In addition, sympathetic innervation is more sensitive than myocardial muscle cells to damage caused by ischemia, rendering mismatched myocardial areas. The preserved viability in a denervated myocardium could create an ideal substrate for arrhythmogenicity that would lead to lethal arrhythmic events [26]. Such a characteristic mismatch between myocardial innervation and perfusion is often noticeable within the intermediate range of H/M ratio in patients with ischemic etiology (Fig. 4C). At the lower end of the ^123^I-*m*IBG spectrum (severely denervated myocardium), arrhythmias cannot be induced because of a lack of viable innervation with arrhythmogenicity. On the other hand, a direct sympathetic-innervation-driven substrate for arrhythmias does not exist in a normally innervated myocardium with greater ^123^I-*m*IBG activity. Thus, an intermediate reduction in the ^123^I-*m*IBG H/M ratio associated with increased risk for lethal ArEs reflects the substrate-driven mechanism of pathophysiological sympathetic innervation.

Increased ^123^I-*m*IBG myocardial WRs are also associated with a bell-shaped risk for lethal ArEs. Accelerated WRs of ^123^I-*m*IBG from hearts generally reflects increased sympathetic drive, probably leading to a risk of sudden cardiac death [8]. In addition to the super-sensitivity of adrenoceptors in denervated myocardium, a hyperactive sympathetic drive is likely to exacerbate the increased arrhythmogenicity responsible for lethal ArEs.

### Bell-shape trends assessed by ML models

We introduced multivariate statistical and ML models that predicted prognoses more accurately than basing them on a single variable [12, 15]. Whereas the statistical model predicted overall mortality including HFD and lethal ArEs, the ML model specifically excelled in predicting ArEs. The ML model has the advantage of simulating many combinations of variables to reveal their impact on outcomes (Figs. 6 and 7). We found a distinct bell-shaped curve in NYHA classes II and III, but not in class I. Moreover, the peak was shifted to the right in class III compared with class II. This might partially explain the bell-shaped curves in the ADMIRE-HF and European studies [9–11], while the occurrence of ArEs increases among Japanese patients with a lower H/M range [5, 7, 8]. Although the eGFR was lower in the J than the E group, it was not a determinant of, and the LVEF was not associated with the bell-shaped curve.

These findings have significant clinical implications. When attempting to use the ^123^I-*m*IBG-based risk model to identify indications for cardiac devices or other types of treatments, simple dichotomous classifications such as high and low H/M ratios based on a cutoff of 1.6 might be insufficient. Instead, an appropriate multivariable ML model could be promising to differentiate outcomes into high, intermediate, and low-risk profiles more precisely in terms of lethal arrhythmic events and HFD.

### Limitations

Discrimination and calibration are crucial factors in evaluations of ML-based prediction models [27]. However, we encountered a limitation of the ML model in predicting actual incidences of ArE among European patients. This issue arose because the ML model was originally developed based on data from studies of Japanese patients and clinical settings that aimed to differentiate between patient risk of HFD and ArEs [15]. Tables 1 and 2 show significant differences between groups E and J with respect to NYHA class, renal function, and complications associated with anemia, ischemic etiology of HF, and hypertension. The patients in group E were primarily targeted candidates for cardiac device implantation, which skewed the baseline features towards a moderate or higher risk for ArE. The actual ArE rate in the derivation cohort used to develop the ML model was < 10%. In contrast, HFD data from group E were not available, likely due to selection bias because patients were specifically chosen for cardiac device implantation, and ICDs/CRTDs were implanted in two-thirds of them. This variation in risk probability at the time of initial patient selection could potentially impact the accuracy of the model as shown in the calibration graph, particularly for low-risk patients in Group E.

A recent critical appraisal of artificial intelligence-based models found that low-quality non-representative data within the targeted population could lead to inappropriate predictive performance [28, 29]. Therefore, we used the ML model to characterize the relative contributions of variables such as the H/M ratio, LVEF, and NYHA functional class. In addition, retrospective data collection based on clinical databases, and incomplete information about medications that have progressively advanced, and precise long-term outcomes rendered the results inconclusive. The ML model determined a positive correlation between the predicted ArE rate and the selection of candidates for ICD/CRTD implantation in the J and the E groups. However, while the model effectively predicted the event rate in Japanese patients, further improvements are needed to specifically predict ArEs (%/year) in European clinical practice. Enhancing the model generalization would be possible if it is further trained on a larger and more diverse patient population. Lastly, a high-quality larger database of patients is required to validate the present findings and for future prospective analyses of prognostic interactions among the many variables associated with unfavorable outcomes due to pump failure or lethal arrhythmias.

## Conclusions

The relationship between the ^123^I-*m*IBG H/M ratio and lethal ArE varies according to patient backgrounds and populations. Nevertheless, the bell-shaped curve of lethal ArEs described herein indicated that intermediately impaired cardiac ^123^I-*m*IBG activity, NYHA functional classes II and III, increased WRs, and ischemic etiology are synergistically related to arrhythmogenicity and potential lethal outcomes. Although further investigation is required, a more precise prediction of sudden or arrhythmic death using our method should contribute to a more appropriate and timely selection of preventive strategies to treat patients with CHF.

## Data Availability

The datasets generated and/or analyzed in the current study are not publicly available due to approval of Ethics Committee but are available from the corresponding author on reasonable request.

## Abbreviations

ArE: Arrhythmic event
CHF: Chronic heart failure
CRTD: Cardiac resynchronization therapy with defibrillator
eGFR: Estimated glomerular filtration rate HF, heart failure
HFD: Heart failure death
H/M: Heart-to-mediastinum
ICD: Implantable cardioverter defibrillator
LVEF: Left ventricular ejection fraction
*m*IBG: *Meta*-iodobenzylguanidine
ML: Machine learning
NYHA: New York Heart Association
VT/VF: Ventricular tachycardia or fibrillation
